# The effect of tablet tilt angles and time on posture, muscle activity, and discomfort at the neck and shoulder in healthy young adults

**DOI:** 10.1371/journal.pone.0283521

**Published:** 2023-03-23

**Authors:** Siriyaphorn Rungkitlertsakul, Petcharatana Bhuanantanondh, Bryan Buchholz

**Affiliations:** 1 Faculty of Physical Therapy, Mahidol University, Salaya, Nakhon Pathom, Thailand; 2 Department of Biomedical Engineering, University of Massachusetts Lowell, Lowell, Massachusetts, United States of America; Tokai University, JAPAN

## Abstract

**Background:**

Although young adults regularly perform tablet writing, biomechanics during the tablet writing with different tilt angles has not been studied. The objective of this study was to compare posture, muscle activity, and discomfort at the neck and shoulder between tablet writing with 0° (horizontal) and 30° tablet tilt angles over 40 minutes in healthy young adults.

**Methods:**

Twenty participants wrote continuously for 40 minutes on a tablet with both tilt angles in a randomized order. Between conditions, there was a 5-minute activity break. Differences in neck and shoulder posture, muscle activity, and discomfort between both tablet tilt angles and changes in the outcomes every 10 minutes over 40 minutes were investigated.

**Results:**

With the tilted tablet, there were lower neck flexion (Z = -4.637, P<0.001), lower shoulder extension (Z = -3.734, P<0.001), and lower neck Visual Analogue Scale (VAS) (left; Z = -4.699, P<0.001 and right; Z = -3.874, P<0.001) as compared to the no tilt condition. However, the right upper trapezius muscle activity was higher in the tilted condition as compared to the no tilt one.

Over 40 minutes, the neck VAS (left; χ^2^(4) = 30.235, P<0.001 and right; χ^2^(4) = 32.560, P<0.001) and heart rate variability (χ^2^(4) = 12.906, P = 0.012) showed notable increases after 20 minutes compared to baseline.

**Conclusion:**

In conclusion, adjusting the tablet tilt to 30° and limiting time spent to 20 minutes are recommended for young adults during the tablet writing to prevent neck problems.

## Introduction

Neck and shoulder pain are common health problems related to the use of electronic devices including a tablet [1]. By 2013 to 2017, the popularity of tablet use has been globally increasing from 0.86 to 1.14 billion users [2]. Moreover, a majority of young adult users have experienced body discomfort particularly in the neck and shoulder [3–6]. In addition, repetitive or static neck and shoulder flexion could also contribute to neck and shoulder pain [7].

With the main feature of tablets, the combined screen for data entry and display, resulting in a low viewing angle, users adopted more neck flexion and shoulder elevation during tablet use compared to during computer or laptop use [8]. These awkward postures could increase biomechanical stress at the neck or shoulder. During neck flexion, there were increases of compressive and anteroposterior shear forces at the cervical spine [9] together with greater Cervical Erector Spinae (CES) activation [10,11] that could lead to discomfort [12]. Moreover, greater shoulder flexion was also associated with increased Upper Trapezius (UT) and Anterior Deltoid (AD) muscle activity [13]. Because of repetitive muscle activation of UT and AD with high forces, shoulder discomfort can occur [14].

By tilting a tablet up from horizontal, neck flexion could be reduced because it elevated viewing angles [4,15–18] but shoulder flexion may be increased [19]. Moreover, some tablet users also reported shoulder discomfort at high tablet tilt angles [4,16,20]. Hence, the extreme tablet tilt angles which were 0° (horizontal) and 60° should be avoided to prevent neck and shoulder discomfort, respectively [16]. Additionally, many tasks, e.g., reading, gaming, and typing, were provided in the previous studies [4,15,16,20] but writing was not, even though it was a common task in young adults.

Prolonged time spent on electronic devices could cause localized muscular fatigue [21,22] and postural changes [23]. Not surprisingly, body discomfort also increased with time [24] and that a notable increment was found after 30–45 minutes of sitting [25]. However, previous studies relating to the effect of tablet tilt angles were conducted for short periods. Consequently, 40 minutes, 10 minutes extended from 30 minutes, was used in the current study.

The current research gaps were the limited understanding about how tablet tilt angles and the prolonged tablet writing influenced the neck and shoulder. The current study examined these gaps to clarify 1) if a 30° tablet tilt angle would be beneficial to prevent neck and shoulder problems during tablet writing, and 2) when tablet users should take a break. Therefore, the current study was aimed to compare posture, muscle activity and discomfort at the neck and shoulder between tablet writing with 0° and 30° tablet tilt angles over 40 minutes in healthy young adults. For the current hypotheses, there would be significant differences in the outcomes between 0° and 30° tablet tilt angles as well as among over the 40-minute task duration broken into four-time intervals. Ultimately, the findings would promote the comprehensive development of an ergonomic guideline for tablet users who are young adults.

## Methods

### Participants

Participants were screened about neck and shoulder problems by using the modified Nordic musculoskeletal questionnaire before attending the study. The inclusion criteria were defined as follows; 1. young adults (age ranged 18–25 years), 2. right-hand dominant, 3. at least one year of experience with using a tablet, 4. normal or corrected normal vision, 5. regular use of a tablet of at least 2 hours/day, and 6. no pain in the neck and upper extremities during the past 7 days. Participants were excluded if they had any of these conditions; 1. an accident involving injury of the neck and/or upper extremities within 12 months of the study, 2. history of systematic diseases, 3. history of neurological problems, 4. history of cardiovascular diseases, 5. allergy to rubbing alcohol, and 6. unable to communicate in Thai. All participants signed an inform consent prior to enrolling in the study. The study protocol was approved by the Mahidol University Central Institutional Review Board (MU-CIRB 2021/204.2604).

### Instrument

#### Inertial Measurement Unit (IMU)

The IMU (Trigno^TM^, Delsys Inc., USA) provides body joint kinematic data and was developed by the fusion of accelerometers, gyroscopes, and magnetometers to increase data accuracy. The IMU provided acceptable accuracy compared to the 3D motion system [26,27] and moderate to excellent test-retest reliability (ICC = 0.75–0.99) [28].

#### Surface Electromyography (SEMG)

The SEMG (Trigno^TM^, Delsys Inc., USA) is a standard measurement tool for muscle activity. It detects myoelectrical signals from the electrodes placed on the skin over the investigated muscles. The size and weight of a SEMG and IMU sensor in combination (Trigno Avanti^TM^, Delsys Inc., USA) are 27*37*13 mm and 14 g.

#### Visual Analogue Scale (VAS)

VAS is a measurement of pain intensity rated by participants and ranges from 0 (no pain) to 10 (worst possible pain). The electronic VAS via the Interactive Clinics application had high inter-method reliability (ICC = 0.94, 95%CI = 0.91–0.96) compared to the traditional paper-based VAS and excellent intra- method reliability (ICC = 0.94, 95%CI = 0.91–0.96) [29].

#### Heart Rate Variability (HRV)

Heart rate variability (HRV; Polar H7 heart rate sensor, Polar Inc., NY) is a measure of the fluctuations in interbeat intervals and is influenced by the autonomic nervous system. Previously, HRV was applied as an objective assessment of pain responses [30]. The concurrent validity of HRV collected via a smart phone application was excellent compared to the electrocardiogram (r = 0.85–0.99) [31].

### Procedures

**Fig 1:**
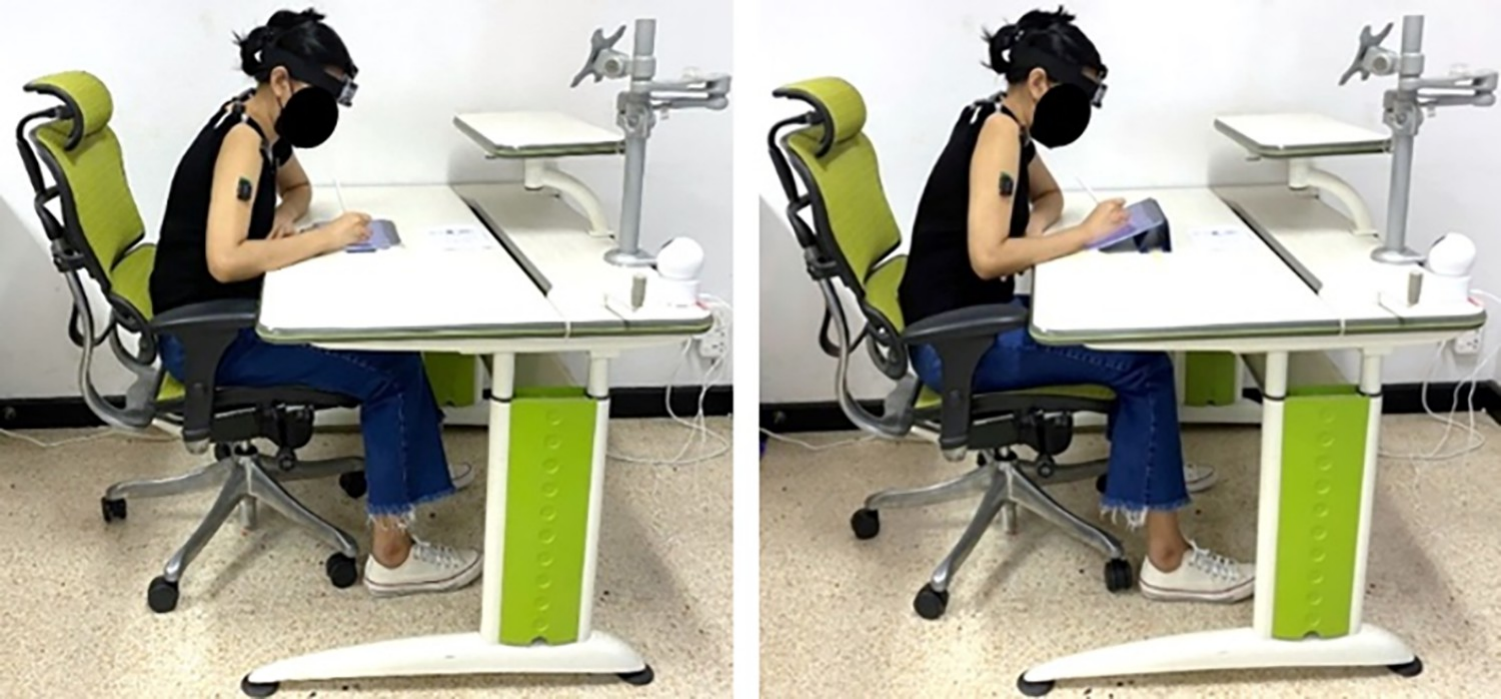
Tablet writing with the 0° (left) and 30° tilt angles (right).

This study was a cross-sectional study with a repeated-measures design comparing neck and shoulder posture, muscle activity, and discomfort between tablet tilt angles (0° and 30°) as shown in Fig 1 and among time intervals (0-10^th^ minute, 11^th^-20^th^ minute, 21^st^-30^th^ minute, and 31st– 40^th^ minute). The experiment was conducted in the morning (9.00 a.m.– 12.00 p.m.) to avoid fatigue from studying or working.

#### Workstation set-up

A table and a chair were adjusted to fit each participant’s anthropometry. The chair height was adjusted for participants’ thighs being parallel to the ground and feet being flat on the ground [32] while the table height was at 5 cm above the resting-elbow level [33]. A tablet (iPad Pro 2020 with 2^nd^-generation Apple Pencil, Apple Inc., USA) was placed on the adjusted table by setting its bottom screen 10 cm away from and parallel to the edge of the table [33].

#### Outcome measurement

The IMU sensors were applied at the middle of forehead and at the middle of the humerus on the right side. Before the application of SEMG, participants’ hair over the areas of sensor placement was removed and their skin was cleaned with alcohol. The SEMG sensors were attached to the participants’ bodies following the European recommendations for SEMG [34]. To measure bilateral CES muscle activity, the sensors were placed 2 cm lateral to the spinous process of the 4^th^ cervical vertebra along the direction of muscle fibers. To measure bilateral UT muscle activity, the sensors were placed at the mid-point between the acromion process and the spinous process of the 7^th^ cervical vertebra. To measure right AD muscle activity, the sensor was placed 2 cm away from the anterior edge of the muscle and 3 cm. below the anterior rim of the acromion process. The polar heart rate sensor was moistened and firmly attached below the chest muscles of participants to measure objective discomfort. VAS, on the application called Interactive Clinics, was rated by participants to measure subjective discomfort in the neck and shoulder.

At the beginning of each experimental condition, baseline data for all measures were collected. To collect the baseline of the IMUs and SEMG data, participants were instructed to sit on an adjusted chair while keeping their neck and trunk straight and arms at their sides for one minute. To collect the baseline for the HRV data, they sat comfortably on the chair using the backrest for 5 minutes. Finally, they were asked to rate the baseline discomfort with the VAS.

During each 10-minute interval over the 40-minute writing task, linear acceleration measured by IMU, and muscle activity measured by SEMG were recorded at the beginning (2^nd^ -3^rd^ minute), middle (5^th^ -6^th^ minute), and last (8^th^ -9^th^ minute) segments of the interval. The data for the three segments were averaged to represent the data for that interval. Moreover, the HRV and VAS data were recorded during the last 5 minutes of each interval.

#### Experimental description

Participants wrote on the tablet with both tilt angles continuously for 40 minutes. Between tablet tilt angles, there was a 5-minute activity break where participants stood and stretched their bodies following the instruction from the audio clip [35]. For the writing condition, participants were provided with a paragraph from Aesop’s fables in Thai language containing 60–80 words that they re-wrote in the blank space below the paragraph at their comfortable writing pace. During the experiment, participants were not allowed to move the tablet, zoom in/out or change pencil colors to provide similarity of the task for all participants. They sat in a comfortable posture but were instructed not to lean on the backrest to avoid physical contact with the SEMG and IMU sensors. In addition, they were asked to avoid movement not related to the writing task, for instance, body stretching.

### Data analysis

To process IMU data, the 0.2-second moving average was performed to smooth linear acceleration respecting to the X, Y, and Z axes (a_x_, a_y_, and a_z_). Neck and shoulder angles in the sagittal plane were calculated from two relative vectors by the formula “neck flexion or extension = tan^-1^(a_z_ / a_y_)” and “shoulder flexion or extension = tan^-1^(a_x_ / a_y_)”, respectively. Positive values identified flexion, while negative ones were extension.

Raw EMG signals were collected with a sampling frequency of 1200 Hz and band-pass filtered (20–450 Hz). To convert the raw EMG signals to Root Mean Square (RMS), the signals were corrected for DC offset, rectified, and low-pass filtered with a Butterworth (2^nd^ order, 20 Hz cutoff frequency) via the EMGworks® Analysis Software for PC (Delsys Inc., USA). The EMG signals were normalized by the maximal observed signal of each muscle across both experimental conditions over the 80 minutes of data collection.

The HRV data were analyzed by performing the medium artefact correction with 5% acceptance threshold and 500-lamba smoothness priors via the Kubios HRV standard software (Kubios Oy, Finland). For the ratio of low frequency and high frequency (LF/HF), the spectrum estimation was obtained by the Welch’s periodogram method.

### Statistical analysis

The SPSS program version 22 (IBM SPSS Statistics V22.0 for Windows, SPSS Inc., USA) was used for all statistical analyses. The descriptive statistics was used to analyze the demographic data as well as the median and interquartile range (IQR) of outcomes. The Shapiro-Wilk test was used to test for the normality of the data. If the data were found to not have a normal distribution, the Wilcoxon signed-rank test and the Friedman’s test were used to investigate differences between tablet tilt angles and among time intervals, respectively. The significant level was set at α = 0.05 for the primary analyses. If a significant difference among time intervals was found, pairwise comparisons by the Wilcoxon signed-rank test would be conducted. The adjusted significance level for the 10 pairwise comparisons was set at α = 0.005.

## Results

A total of 20 young adults, consisting of 2 males and 18 females, participated in this study (mean ± standard deviation, age: 20.30 ± 2.13 years, weight: 56.88 ± 10.48 kg., and height: 162.28 ± 6.40 cm.). Of the 20 participants, 13 (65%) and 7 (35%) regularly adjusted tablet tilt angles for 0° and 20°-35°, respectively during writing. Moreover, 11 (55%) of them preferred placing a tablet screen parallel to an edge of a table whereas 9 (45%) preferred rotating a tablet screen during writing.

The baseline comparisons of all outcomes showed no significant differences between 0° and 30° tablet tilt angles as presented in Table 1.

**Table 1:** The baseline comparison of outcomes between tablet tilt angles.

Outcomes				Tablet tilt angles		
				Median (IQR)		P-value
				0° tablet tilt angle	30° tablet tilt angle	
Posture (°) Flexion (+)/Extension (-)	Neck			-6.105 (6.695)	-6.205 (4.828)	0.240
	Shoulder			-1.345 (4.320)	-1.595 (3.113)	0.331
**Muscle activity (Normalized EMG)**	CES		Left	0.093 (0.071)	0.101 (0.066)	0.616
			Right	0.063 (0.035)	0.069 (0.037)	0.731
	UT		Left	0.027 (0.052)	0.027 (0.051)	0.491
			Right	0.016 (0.015)	0.016 (0.016)	0.396
	Right AD			0.029 (0.026)	0.028 (0.020)	0.432
**Discomfort**	VAS	Neck	Left	0.000 (0.000)	0.000 (0.000)	1.000
			Right	0.000 (0.000)	0.000 (0.000)	1.000
		Shoulder	Left	0.000 (0.000)	0.000 (0.000)	1.000
			Right	0.000 (0.000)	0.000 (0.000)	1.000
	HRV (LF/HF)			0.660 (0.300)	0.600 (0.720)	0.820

IQR = Interquartile Range, CES = Cervical Erector Spinae, UT = Upper Trapezius, AD = Anterior Deltoid, VAS = Visual Analogue Scale, HRV = Heart Rate variability and LF/HF = Ratio of low frequency and high frequency.

### The effect of tablet tilt angles

The effect of tablet tilt angles is shown in Table 2.

**Table 2:** The effect of tablet tilt angle on neck and shoulder posture, muscle activity, and discomfort.

Outcomes			Tablet tilt angles			
			Median (IQR)			P-value
			0° tablet tilt angle		30° tablet tilt angle	
Posture (°) Flexion (+)/Extension (-)	Neck		30.355 (16.785)		27.870 (15.780)	<0.001*
	Shoulder		-9.255 (14.110)		-4.770 (10.283)	<0.001*
**Muscle activity (Normalized EMG)**	CES	Left	0.170 (0.114)		0.176 (0.112)	0.928
		Right	0.148 (0.114)		0.152 (0.115)	0.252
	UT	Left	0.046 (0.049)		0.050 (0.050)	0.943
		Right	0.062 (0.066)		0.078 (0.075)	<0.001*
	Right AD		0.041 (0.026)		0.041 (0.025)	0.554
**Discomfort**	VAS	Neck	Left	0.000 (1.385)	0.000 (0.000)	<0.001*
			Right	0.000 (1.100)	0.000 (0.000)	<0.001*
		Shoulder	Left	0.000 (0.000)	0.000 (0.000)	0.875
			Right	0.000 (0.000)	0.000 (0.000)	0.657
	HRV (LF/HF)		1.140 (1.020)		1.120 (1.420)	0.592

IQR = Interquartile range, EMG = Electromyography, CES = Cervical Erector Spinae, UT = Upper Trapezius, AD = Anterior Deltoid, VAS = Visual Analogue Scale, HRV = Heart Rate Variability, LF/HF = Ratio of low frequency and high frequency, and

* P <0.05.

#### Posture

Neck flexion (Z = -4.637, P<0.001) and shoulder extension (Z = -3.734, P<0.001) at the 0° tablet tilt angle were significantly higher than those at the 30° tablet tilt angle.

#### Muscle activity

The right UT amplitude at the 30° tablet tilt angle significantly increased compared to the other tilt angle (Z = -3.820, P < 0.001). Nevertheless, there was no significant differences in bilateral CES, the left UT and the right AD amplitudes between both tablet tilt angles.

#### Discomfort

The 0° tablet tilt angle induced significantly more neck VAS compared to the other tilt angle (Left; Z = -4.699, P <0.001 and right; Z = -3.874, P <0.001), whereas shoulder VAS and LF/HF showed no significant differences between tablet tilt angles.

### The effect of time spent

#### Posture

Among time intervals, there were significant differences in neck flexion (χ^2^(4) = 80.380, P<0.001) and shoulder extension (χ^2^(4) = 30.820, P<0.001). Nevertheless, these significant differences were found only between the baseline and each of four intervals (neck flexion; Z = -5.511, P<0.0001, and shoulder extension; Z = -4.234 to -3.683, P <0.0001 to 0.0002), but not among the four intervals shown in Table 3.

**Table 3:** The effect of time on neck and shoulder posture and muscle activity.

Outcomes			Time intervals						
			Median (IQR)					P-value	
			Baseline	1^st^	2^nd^	3^rd^	4^th^	Primary analyses	Pairwise comparisons
Posture (°) Flexion (+) /Extension (-)	Neck		-6.110 (5.550)	31.305 (13.680)	29.960 (9.888)	31.495 (11.565)	30.875 (9.878)	<0.001*	1^st^ & 2^nd^ = 0.851 1^st^ & 3^rd^ = 0.930 1^st^ & 4^th^ = 0.707 2^nd^ & 3^rd^ = 0.952 2^nd^ & 4^th^ = 0.767 3^rd^ & 4^th^ = 0.888
	Shoulder		-1.510 (3.325)	-10.440 (14.888)	-8.665 (11.903)	-9.875 (12.498)	-9.025 (12.810)	<0.001*	1^st^ & 2^nd^ = 0.211 1^st^ & 3^rd^ = 0.245 1^st^ & 4^th^ = 0.333 2^nd^ & 3^rd^ = 0.199 2^nd^ & 4^th^ = 0.762 3^rd^ & 4^th^ = 0.460
**Muscle activity (Normalized EMG)**	CES	Left	0.100 (0.068)	0.194 (0.110)	0.195 (0.111)	0.194 (0.121)	0.194 (0.101)	<0.001*	1^st^ & 2^nd^ = 0.105 1^st^ & 3^rd^ = 0.029 1^st^ & 4^th^ = 0.032 2^nd^ & 3^rd^ = 0.398 2^nd^ & 4^th^ = 0.402 3^rd^ & 4^th^ = 0.919
		Right	0.064 (0.035)	0.163 (0.085)	0.162 (0.070)	0.159 (0.076)	0.166 (0.072)	<0.001*	1^st^ & 2^nd^ = 0.753 1^st^ & 3^rd^ = 0.063 1^st^ & 4^th^ = 0.044 2^nd^ & 3^rd^ = 0.030 2^nd^ & 4^th^ = 0.014 3^rd^ & 4^th^ = 0.753
	UT	Left	0.027 (0.042)	0.052 (0.059)	0.055 (0.052)	0.050 (0.046)	0.048 (0.045)	<0.001*	1^st^ & 2^nd^ = 0.834 1^st^ & 3^rd^ = 0.167 1^st^ & 4^th^ = 0.258 2^nd^ & 3^rd^ = 0.350 2^nd^ & 4^th^ = 0.255 3^rd^ & 4^th^ = 0.532
		Right	0.016 (0.016)	0.087 (0.060)	0.079 (0.050)	0.074 (0.060)	0.080 (0.059)	<0.001*	1^st^ & 2^nd^ = 0.624 1^st^ & 3^rd^ = 0.546 1^st^ & 4^th^ = 0.501 2^nd^ & 3^rd^ = 0.149 2^nd^ & 4^th^ = 0.328 3^rd^ & 4^th^ = 0.845
	Right AD		0.028 (0.023)	0.044 (0.025)	0.043 (0.025)	0.041 (0.028)	0.042 (0.033)	<0.001*	1^st^ & 2^nd^ = 0.971 1^st^ & 3^rd^ = 0.065 1^st^ & 4^th^ = 0.560 2^nd^ & 3^rd^ = 0.088 2^nd^ & 4^th^ = 0.771 3^rd^ & 4^th^ = 0.631

IQR = Interquartile range, EMG = Electromyography, CES = Cervical Erector Spinae, UT = Upper Trapezius, AD = Anterior Deltoid, 1^st^ = 1^st^ interval, 2^nd^ = 2^nd^ interval, 3^rd^ = 3^rd^ interval, and 4^th^ = 4^th^ interval, and

* P <0.05 (Significant difference for primary analyses).

#### Muscle activity

There were significant changes in CES (left and right; Z = -5.498 to -5.511, P < 0.0001), UT (left; Z = -4.083 to -4.557, P < 0.0001 and right; Z = -5.498, P < 0.0001), and AD (right; χ^2^(4) = 47.692, P<0.001) amplitudes between the baseline and each time interval, but not among the four intervals shown in Table 3.

#### Discomfort

**Fig 2:**
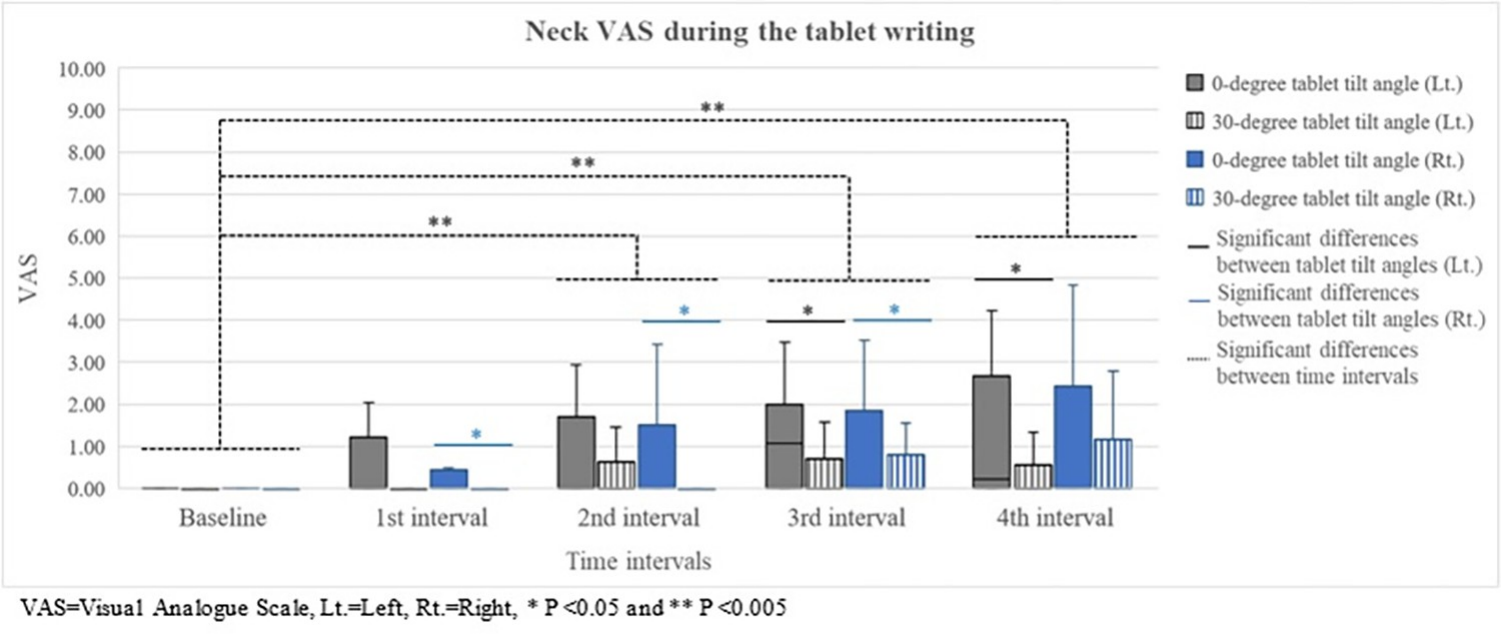
Neck VAS during the tablet writing.

**Fig 3:**
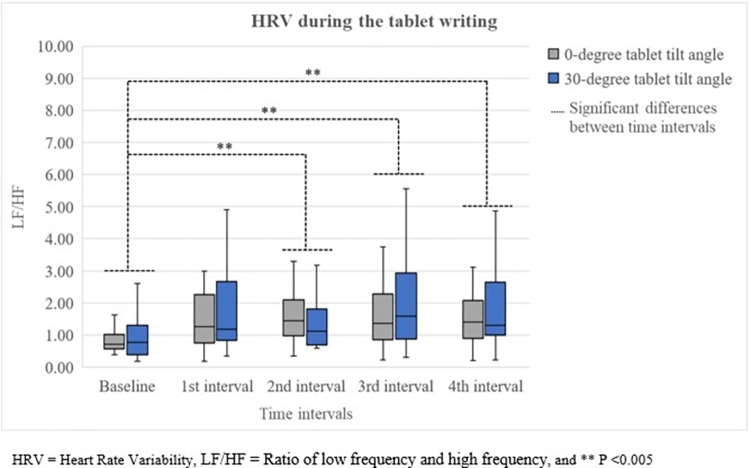
HRV during the tablet writing.

Neck VAS revealed a significant increasing trend over 40 minutes (left; χ^2^(4) = 30.235, P<0.001 and right; χ^2^(4) = 32.560, P<0.001) as shown in Fig 2. In addition, the first notable increase was found at 20 minutes (left; Z = -3.180, P = 0.001 and right; Z = -2.934, P = 0.003). However, there were no significant differences in shoulder VAS among time intervals. For the LF/HF, those in 20, 30, and 40 minutes significantly increased from the baseline (χ^2^(4) = 12.906, P = 0.012) presented in Fig 3.

## Discussion

### Posture

The current finding showed neck flexion at the 0° tablet tilt angle was larger than that at the 30° one but there were no significant changes in neck flexion at both tablet tilt angles over 40 minutes. Between tablet tilt angles, decreased neck flexion with the increased tablet tilt angle was consistent with previous studies [4,15,16]. However, neck flexion at both tablet tilt angles in the current study still exceeded 20°, the cut-off value for low risk of neck pain [36]. This could be interpreted that only adjusting the 30° tablet tilt angle is insufficient to prevent neck problems. Constant neck flexion over 40 minutes was not in line with the study of Szeto et al. [23]. They found the decreasing tendency of neck flexion over the 30-minute tablet reading [23]. The inconsistent finding might result from different posture during tablet use. According to the study of Szeto et al., participants were allowed to vary postures naturally as the current study; however, they were instructed to hold the tablet with both hands, while participants in the current study were instructed to place the tablet on a table. Compared to placing a tablet and arms on a table, holding a tablet with hands provided larger neck flexion and more changes in neck position [37]. Although there was not a gradual change of neck posture across the time, rapid postural changes, including neck extension or trunk straightening, occurred during the tablet writing. However, these postural changes lasted only a few seconds. Hence, the median neck flexion showed no differences among time intervals.

For shoulder posture, shoulder extension at the 30° tablet tilt angle was greater than that at 0°; moreover, shoulder posture at both tablet tilt angles remained steady over 40 minutes. On the contrary, Young et al. discovered that tablet users flexed their shoulders more during tablet use with the increased tilt angle [19]. Possible causes for subjects tending to perform shoulder extension rather than shoulder flexion in the current study might be different tablet location, no backrest use, and no tablet screen rotation. For tablet locations, participants in the study of Young et al. placed a tablet on a table and a lap when the tablet tilt angles were adjusted for 45° and 15°, respectively. Placing the tablet on a lap possibly reduced shoulder flexion because it kept the tablet close to the body. Nevertheless, a tablet was placed on a table for both tablet tilt angles in this study. For use of the backrest, it was allowed in the study of Young et al. but not in the current study to avoid physical contacts on the SEMG sensors. With restriction of using the backrest, participants leaned forward which possibly enhanced shoulder extension because forward leaning could reduce distance between participants’ bodies and the tablet screen. Moreover, the tablet screen was fixed with no screen rotation in the current study. With no tablet screen rotation, participants, who were familiar with the tablet writing with screen rotation, rotated their trunk instead of flexing their shoulder. Over 40 minutes, shoulder extension did not change. According to previous studies, there was limited knowledge of shoulder postural changes across the prolonged tablet use. Hence, this finding provided the information that time spent could not influence shoulder extension during the tablet writing with the writing upper extremity being supported.

### Muscle activity

Similar amplitudes for CES muscle activity between tablet tilt angles and among time intervals were found. Between tablet tilt angles, Intolo and Plangsiri found similar findings that there was no significant difference of CES muscle activity between 20-minute tablet use with and without a tilt angle in female office workers [17]. However, another study by Intolo et al. showed that the 60° tablet tilt angle decreased the CES activation of children during gaming for 15 minutes when compared to no tilt angle [18]. This inconsistency possibly resulted from different amounts of tablet tilt and groups of samples. Compared to the 30° tablet tilt, the 60° one certainly raises a tablet screen closer to the eye level and reduces neck flexion. Moreover, CES muscle activities for children tended to have larger amplitudes during neck flexion compared to adults because their larger relative proportion of head to trunk lead to the increased load at the neck [38]. Additionally, the CES muscle activity was constant over the 40-minute tablet writing. This was inconsistent with the study of Szeto et al. that found increased CES muscle activity over the 30-minute tablet reading [23]. Constant CES muscle activity found in the current study possibly resulted from neck movement during the tablet writing. Participants extended their neck from time to time to relieve discomfort. Apart from neck extension, participants also performed neck lateral flexion to the left and right alternately to avoid the tablet screen being blocked by the writing hand. These neck movements might cause inconsistent muscle activation and the increase of blood flow which might prevent muscular fatigue.

The 30° tablet tilt angle induced a larger amplitude of the right UT muscle activity but not the left one compared to the 0° tablet tilt angle; moreover, UT did not differ over 40 minutes. Between tablet tilt angles, the current finding did not correspond with the study of Chiu et al. [20]. They found the UT muscle activity increased with increased tablet tilt angles [20]. This inconsistency could be explained by different table heights. The table was 3 cm below resting elbow level in the study of Chiu et al. and 5 cm above in the current study. Table height influenced the tablet screen height. Either low or high tablet screen could increase UT muscle activity. For a low tablet screen, UT muscle activity compensated with larger activation to hold the head during deep neck flexion. For a high tablet screen, UT muscle activity increased due to increased height of working surface. Over 40 minutes, both UT muscle activity remained steady. This probably resulted from arm support [13]. Because of arm support, the UT muscles perform intermittently and with decreased amplitude during writing. Accordingly, load was insufficient to induce muscle fatigue.

Both tablet tilt angles and time spent did not affect the right AD muscle activity. This result was inconsistent with the study of Chiu et al. which found that AD muscle activity increased with increased tablet tilt angles [20]. Similarly to the UT activity, the possible reason was arm support [13].

### Discomfort

The 30° tablet tilt angle could reduce neck discomfort compared to the other tilt angle; however, this was inconsistent with the previous study that VAS discomfort was not significantly different between tablet tilt angles [20]. The possible reason was short duration of tablet use (15 minutes). This duration might not have been long enough to induce discomfort. Additionally, neck discomfort tended to increase over 40 minutes while the first notable increase from the baseline was at 20 minutes. This was consistent with the study of Intolo and Plangsiri [17]. They found that neck discomfort significantly increased from the baseline after 20 minutes of tablet use [17]. Although the significant increase of neck VAS with increasing time was found, the differences were not clinically meaningful. To illustrate, the differences of left and right neck VAS between at the baseline and at 40 minutes were 0.738 and 0.850 respectively. These were lower than 1.700 (the minimal clinically important difference of VAS) [39].

Shoulder discomfort did not alter between either tablet tilt angles or time intervals. This was consistent with the study of Chiu et al. which found similar shoulder comfort among tablet tilt angles [20]. However, the duration of tablet use was only 15 minutes. Moreover, comparisons of shoulder discomfort over time during tablet use were still limited. For the current study, no change of shoulder discomfort over the prolonged tablet writing was possibly due to shoulder posture minimally deviating from neutral. Also, low AD muscle activity was found in both tablet tilt angles over 40 minutes, as compared to the baseline.

For HRV, the LF/HF showed no significant difference between tablet tilt angles. This finding was inconsistent with the studies of Le and Marras [40], and Weston et al. [41]. Le and Marras found that there were significant differences of the LF/HF among seated, standing, and perching [40]. The possible reason of the inconsistency was minimal postural differences between both tablet tilt angles. With slight differences in writing posture, biomechanical and muscular load differences might be insufficient to cause physiological discomfort between tilt angles. Weston et al. discovered that the HRV could differentiate discomfort between chairs with fixed and flexible back rest [41]. Although only seating was investigated in the study of Weston et al. which was similar to the current study, VAS at all discomfort areas (neck, upper back, lower back, buttocks, wrists and hands). VAS at the chair with fixed backrest were more than those at the chair with flexible backrest. However, in the current study, VAS at all discomfort areas was in contrast between both tablet tilt angles. Between tablet tilt angles, a significantly higher neck VAS was found at the 0° tablet tilt angle whereas the 30° tablet tilt angle induced significantly more VAS at the right elbow, wrist, and hand.

Over 40 minutes, the LF/HF showed notable increases from the baseline at 20 minutes which was consistent with the result of neck VAS. However, there was no change of LF/HF over 40 minutes of writing. This was consistent with the study of Weston et al. [41] but not consistent with the study of Le and Marras [40]. Weston et al. found that the LF/HF did not alter whereas Le and Marras reported the increasing trend of the LF/HF over an hour. However, the increase of the LF/HF during the seating condition was minimal. The inconsistent finding with the study of Le and Marras might result from different age ranges of participants and length of spent time. Participants in the study of Le and Marras (Mean age = 26 ±.8.5 years) were older than those in the current study (Mean age = 20.3 ± 2.1 years). Moreover, time spent on the tablet in the study of Le and Marras (an hour) was longer than that in the current study (40 minutes). With younger participants and shorter duration, biomechanical load is not accumulated enough to notably increase discomfort and change the LF/HF.

Overall, tablet users had less neck flexion and shoulder extension during writing on the tablet with a 30° tilt angle as compared to no tilt angle. With less neck flexion, neck discomfort will be reduced due to decreased load on the cervical spine. Moreover, lower neck flexion at the tilted tablet should have reduce UT and CES muscle activity as in previous studies [42,43]. However, lower neck flexion did not decrease either UT or as CES muscle activity. The lack of a difference in UT and CES muscle activity was a result of the small differences in neck flexion between tablet tilt angles. Additionally, increased right UT muscle activity was mainly influenced by the elevated working surface as opposed to neck flexion. Although the increased tablet tilt angle resulted in less shoulder extension, AD muscle activity and shoulder discomfort were similar between both tilt angles. This was related to the arm support that was provided to participants. Over 40 minutes, steady neck and shoulder postures were associated with no change in CES, UT and AD muscle activity. Nevertheless, neck discomfort increased with time because load accumulated on tissues during sustain neck flexion leading to neck discomfort.

### Strengths and limitations

The main strength of the current study was the investigation of comprehensive outcomes for both biomechanical and physiological aspects during extended duration of the tablet writing. However, this study also had limitations; 1. Neck and posture were observed only in the sagittal plane. However, participants seemed to frequently move the neck in the frontal and transverse planes as well. 2. All participants in the current study were healthy young adults; thus, the current finding cannot be applied to all tablet users. Further studies should observe neck posture in other planes to investigate asymmetrical posture which might increase risk of having neck pain. In addition, different groups of participants, such as older adults or participants with neck-shoulder pain, should be studied because degeneration [44] or altered motor control patterns [45,46] may lead to contrast results.

## Conclusion

The 30° tablet tilt angle could reduce neck flexion, shoulder extension, and discomfort. However, the right UT muscle activity functioned with larger amplitude at this angle. Additionally, notable increases from the baseline of neck VAS and HRV were firstly found in the 20 minutes.

## Supporting information

S1 File (XLSX)
